# Detecting failure modes in image reconstructions with interval neural network uncertainty

**DOI:** 10.1007/s11548-021-02482-2

**Published:** 2021-09-04

**Authors:** Luis Oala, Cosmas Heiß, Jan Macdonald, Maximilian März, Gitta Kutyniok, Wojciech Samek

**Affiliations:** 1 Department of Artificial Intelligence, Fraunhofer HHI, Berlin, Germany; 2 Institut für Mathematik, Technische Universität Berlin, Berlin, Germany; 3 Mathematisches Institut, Ludwig-Maximilians-Universität München, Munich, Germany

**Keywords:** Deep learning, Image reconstruction, Uncertainty quantification, Failure modes

## Abstract

**Purpose:**

The quantitative detection of failure modes is important for making deep neural networks reliable and usable at scale. We consider three examples for common failure modes in image reconstruction and demonstrate the potential of uncertainty quantification as a fine-grained alarm system.

**Methods:**

We propose a deterministic, modular and lightweight approach called Interval Neural Network (INN) that produces fast and easy to interpret uncertainty scores for deep neural networks. Importantly, INNs can be constructed post hoc for already trained prediction networks. We compare it against state-of-the-art baseline methods (MCDrop, ProbOut).

**Results:**

We demonstrate on controlled, synthetic inverse problems the capacity of INNs to capture uncertainty due to noise as well as directional error information. On a real-world inverse problem with human CT scans, we can show that INNs produce uncertainty scores which improve the detection of all considered failure modes compared to the baseline methods.

**Conclusion:**

Interval Neural Networks offer a promising tool to expose weaknesses of deep image reconstruction models and ultimately make them more reliable. The fact that they can be applied post hoc to equip already trained deep neural network models with uncertainty scores makes them particularly interesting for deployment.

## Introduction

The reconstruction of unknown signals from indirect measurements plays an important role in many applications, including medical imaging [2, 14]. Typically, such tasks are modeled as finite-dimensional linear inverse problems

1 $$\begin{aligned} \varvec{y}= \varvec{A}\varvec{x}+ \varvec{\eta }, \end{aligned}$$

where $\varvec{x}\in \mathbb {R}^n$ is the signal of interest, $\varvec{A}\in \mathbb {R}^{m\times n}$ denotes the forward operator representing a physical measurement process, and $\varvec{\eta }\in \mathbb {R}^m$ is modeling noise in the measurements. Important examples include magnetic resonance imaging and computed tomography, where $\mathbf {A}$ is a subsampled discrete Fourier or Radon transform, respectively. Solving the inverse problem (1) requires computing an approximate reconstruction of $\varvec{x}$ from the observed measurements $\varvec{y}$.

Classical reconstruction methods, e.g., based on sparse regularization models, constitute the state of the art for solving (1) in many cases and are backed by theoretical guarantees [8]. Recently, data-driven deep learning methods are increasingly gaining attention and are repeatedly able to outperform traditional solvers in terms of empirical reconstruction performance or speed, see for example [2].

Despite the advantages, the use of deep learning methods in sensitive applications such as clinical diagnosis is still a concern [23], due to questions regarding the reliability and robustness of the obtained reconstructions when compared to traditional approaches [1, 13]. What is more, erroneous artifacts in the reconstructed signals can be hard to detect as they tend to “blend in” well with the rest of the signal.

Various approaches for incorporating uncertainty quantification (UQ) into deep learning have been proposed to address these issues [10, 16, 18, 22]. However, as we demonstrate, existing UQ approaches come with limitations regarding their capacity to detect failure modes or their post hoc applicability to trained deep learning models.

In this work, we consider a straight-forward approach to solving (1) by employing a neural network to post-process a standard model-based inversion as in [14]. This reconstruction is given by

$$\begin{aligned} \varvec{x}_{\texttt {rec}} = \left( \varvec{\varPhi }\circ \varvec{A}^\dagger \right) (\varvec{y}), \end{aligned}$$

where $\varvec{\varPhi }:\mathbb {R}^n\rightarrow \mathbb {R}^n$ is a neural network trained to minimize the loss $\Vert \varvec{x}-\varvec{\varPhi }(\varvec{A}^\dagger (\varvec{y}))\Vert _2^2$ and $\varvec{A}^\dagger :\mathbb {R}^m\rightarrow \mathbb {R}^n$ denotes the non-learned model-based inversion (e.g., the filtered back-projection in the case of Radon measurements). We will denote $\varvec{z}=\varvec{A}^\dagger (\varvec{y})$ in the following. Given $\varvec{y}$ or $\varvec{z}$, a UQ method is supposed to extend the predicted reconstruction $\varvec{\varPhi }(\varvec{z})$ by a component-wise uncertainty score $\varvec{u}(\varvec{z})$ that provides additional information regarding the reliability of the reconstruction. Therefore, $\varvec{u}(\varvec{z})$ should be correlated with the component-wise error $|\varvec{x}-\varvec{\varPhi }(\varvec{z})|$. We evaluate this for three different failure modes [7] that can arise during inference (see “Experiment B (i): general prediction error detection” section to “Experiment B (iii): Atypical Artifact Detection” section for more details):

(i) Errors caused solely by the ill-posedness of (1), which is mostly determined by the strength of measurement noise and the amount of undersampling,
(ii) Errors caused by adversarial perturbations to the network inputs,
(iii) Errors caused by atypical artifacts that have not been seen during the training.

Our main contributions can be summarized as follows: We present a deterministic, modular and fast UQ-method for deep neural networks (DNNs), called Interval Neural Networks (INN). We evaluate INNs for the detection of the three different image reconstruction failure modes and demonstrate that they provide improved results compared to two existing UQ methods.

## Related work

Whereas a number of methods from classical statistical learning theory, such as Gaussian processes and approximations thereof [6, 19], come with built-in uncertainty estimates, DNNs have been limited in this regard. A surge of efforts to treat neural networks from a variational perspective [3, 16] started to change that. In addition, there exist strands of research in deep learning explicitly occupied with the detection of failure modes caused by adversarial and out of distribution (OoD) inputs. These include Maximum Mean Discrepancy, Kernel Density Estimation and other tools, see [5] or the Minimum Covariance Determinant method [26], Support Vector Data Description [28], among others. We refer to [27] for a comprehensive overview. The detection of adversarial and OoD inputs in these works is typically done in the classification setting. We emphasize that image-to-image regression is a fundamentally different task: While classification is inherently discontinuous, image reconstruction addresses a problem that allows for stable solution methods in many cases, e.g., by sparse regularization. Furthermore, we are not interested in a crude, outright rejection of data points in the *input space* but rather seek to obtain fine-grained information about erroneous artifacts in the *output space*. More closely related to our goal is Monte Carlo dropout (MCDrop) [10] and direct variance estimation (ProbOut) [12], where epistemic and aleatoric uncertainty quantification was considered for segmentation and depth-estimation tasks. Hence, we include their approaches as baseline comparison methods, see “Baseline UQ methods” section.

## Methods

**Fig. 1:**
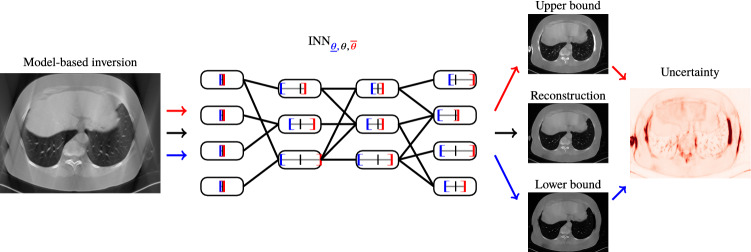
A schematic overview of the proposed Interval Neural Networks for image reconstruction

Popular existing UQ frameworks for DNNs place parametric densities, most commonly Gaussian densities, over the DNN parameters or predictions. Instead of using specific parametrized densities, our INN method relies on bounding distributions using intervals. This results in a flexible and modular method that can be applied post hoc to a given DNN $\varvec{\varPhi }$ that has already been trained. A schematic illustration is provided in Fig. 1: The INN is formed by wrapping additional weight and bias intervals around the weights and biases of the underlying prediction DNN. This allows us to equip the DNN $\varvec{\varPhi }$ with uncertainty capabilities without the need to modify $\varvec{\varPhi }$ itself. After training the INN  we obtain prediction intervals that are guaranteed to contain the original prediction of the underlying network and are easy to interpret. They provide exact upper and lower bounds for the range of possible values that the DNN prediction may take when slightly modifying the network parameters within the prescribed weight and bias intervals.

Previously, the capacity of neural networks with interval weights and biases was evaluated for fitting interval-valued functions [11]. In contrast to [11], our targets $\varvec{x}_i$ are neither interval-valued nor univariate, leading to a different loss function which allows us to equip trained neural networks with uncertainty capabilities *post hoc*. For a direct comparison, see 3 in 3.2 and Equation (18) in [11]. Further, [17, 30] explored neural networks implementing interval arithmetic for robust classifications. However, in their setting, the focus is purely on representing the *inputs* or *outputs* as intervals but not the *weights* and *biases*. In contrast, our proposed INNs determine interval bounds for all network parameters with the goal of providing uncertainty scores for the predictions of an underlying DNN.

### Arithmetic of Interval Neural Networks

We will now give a description of those INN mechanisms that deviate from standard DNNs. The forward propagation of a single input $\varvec{z}$ through a DNN is replaced by the forward propagation of a component-wise interval-valued input $[\underline{\varvec{z}}, \overline{\varvec{z}}]$ through the INN. This can be expressed similarly to standard feed-forward neural networks but using interval arithmetic instead. For interval-valued weight matrices $[\underline{\varvec{W}}, \overline{\varvec{W}}]$ and bias vectors $[\underline{\varvec{b}}, \overline{\varvec{b}}]$, the propagation through the $\ell$-th network layer can be expressed as

2 $$\begin{aligned} \left[ \underline{\varvec{z}}, \overline{\varvec{z}}\right] ^{(\ell +1)} = \varrho \left( \left[ \underline{\varvec{W}}, \overline{\varvec{W}}\right] ^{(\ell )} \left[ \underline{\varvec{z}}, \overline{\varvec{z}}\right] ^{(\ell )}+ \left[ \underline{\varvec{b}}, \overline{\varvec{b}}\right] ^{(\ell )} \right) . \end{aligned}$$

For nonnegative $[\underline{\varvec{z}}, \overline{\varvec{z}}]^{(\ell )}$, for example when using a nonnegative activation function $\varrho$ such as the ReLU in the previous layer, we can explicitly rewrite (2) as

$$\begin{aligned} \overline{\varvec{z}}^{(\ell +1)}&= \varrho \left( \min \left\{ \overline{\varvec{W}}^{(\ell )},\varvec{0}\right\} \underline{\varvec{z}}^{(\ell )} +\max \left\{ \overline{\varvec{W}}^{(\ell )}, \varvec{0}\right\} \overline{\varvec{z}}^{(\ell )}+ \overline{\varvec{b}}^{(\ell )} \right) ,\\ \underline{\varvec{z}}^{(\ell +1)}&= \varrho \left( \max \left\{ \underline{\varvec{W}}^{(\ell )}, \varvec{0}\right\} \underline{\varvec{z}}^{(\ell )}+ \min \left\{ \underline{\varvec{W}}^{(\ell )}, \varvec{0}\right\} \overline{\varvec{z}}^{(\ell )}+ \underline{\varvec{b}}^{(\ell )} \right) , \end{aligned}$$

where the maximum and minimum are computed component-wise. Similarly, for point intervals $\underline{\varvec{z}}^{(\ell )}=\overline{\varvec{z}}^{(\ell )}=:\varvec{z}^{(\ell )}$, for example, as inputs to the first network layer, we can rewrite (2) as

$$\begin{aligned} \overline{\varvec{z}}^{(\ell +1)}&= \varrho \left( \overline{\varvec{W}}^{(\ell )} \max \{ \varvec{z}^{(\ell )},\varvec{0}\}+ \underline{\varvec{W}}^{(\ell )} \min \{ \varvec{z}^{(\ell )},\varvec{0}\}+ \overline{\varvec{b}}^{(\ell )} \right) ,\\ \underline{\varvec{z}}^{(\ell +1)}&= \varrho \left( \underline{\varvec{W}}^{(\ell )} \max \{ \varvec{z}^{(\ell )},\varvec{0}\}+ \overline{\varvec{W}}^{(\ell )} \min \{ \varvec{z}^{(\ell )},\varvec{0}\}+ \underline{\varvec{b}}^{(\ell )} \right) , \end{aligned}$$

regardless of whether $\varvec{z}^{(\ell )}$ is nonnegative or not. Optimizing the INN parameters requires obtaining the gradients of these operations. This can be achieved using automatic differentiation (backpropagation) in the same way as for standard neural networks.

### Training Interval Neural Networks

Let $\varvec{W}^{(\ell )}$ and $\varvec{b}^{(\ell )}$ be the weights and biases of the underlying prediction network $\varvec{\varPhi }$ and let $\overline{\varvec{\varPhi }}:\mathbb {R}^n \rightarrow \mathbb {R}^n$ and $\underline{\varvec{\varPhi }}:\mathbb {R}^n \rightarrow \mathbb {R}^n$ denote the functions mapping a point interval input $\varvec{z}$ to the upper and the lower interval bounds in the output layer of the INN  respectively. Given data samples $\left\{ \varvec{z}_i,\varvec{x}_i \right\} _{i=1}^m$ the INN parameters $[\underline{\varvec{W}}, \overline{\varvec{W}}]^{(\ell )}$ and $[\underline{\varvec{b}}, \overline{\varvec{b}}]^{(\ell )}$ are trained by minimizing the empirical loss

3 $$\begin{aligned}&\sum _{i=1}^{m}\big \Vert \max \{\varvec{x}_i-\overline{\varvec{\varPhi }}(\varvec{z}_i),\varvec{0}\} \big \Vert _2^2 + \big \Vert \max \{\underline{\varvec{\varPhi }}(\varvec{z}_i)-\varvec{x}_i,\varvec{0}\} \big \Vert _2^2\nonumber \\&\quad + \beta \cdot \big \Vert \overline{\varvec{\varPhi }}(\varvec{z}_i)-\underline{\varvec{\varPhi }}(\varvec{z}_i)\big \Vert _1, \end{aligned}$$

subject to the constraints $\underline{\varvec{W}}^{(\ell )}\le \varvec{W}^{(\ell )}\le \overline{\varvec{W}}^{(\ell )}$ and $\underline{\varvec{b}}^{(\ell )}\le \varvec{b}^{(\ell )}\le \overline{\varvec{b}}^{(\ell )}$ for each layer. This way $\underline{\varvec{\varPhi }}(\varvec{z})\le \varvec{\varPhi }(\varvec{z})\le \overline{\varvec{\varPhi }}(\varvec{z})$ is always guaranteed. The first two terms in (3) encourage that the predicted interval $[\underline{\varvec{\varPhi }}(\varvec{z}_i),\overline{\varvec{\varPhi }}(\varvec{z}_i)]$ should contain the target signal $\varvec{x}_i$, while penalizing each component that lies outside with the squared distance to the nearest interval bound. The second term penalizes the interval size, so that the predicted intervals cannot grow arbitrarily large. While a quadratic penalty of the interval size is also possible and leads to similar theoretical bounds as in (4), we choose to minimize the $\ell _1$-norm to make the intervals more outlier inclusive. In addition, the tightness parameter $\beta > 0$ can further tune the outlier-sensitivity of the intervals. This allows for a calibration of the INN uncertainty scores according to an application specific risk-budget. In practice, we found that choosing $\beta$ similar to the mean absolute error of the underlying prediction network yields a good trade-off between coverage [9] and tightness.

### Properties of Interval Neural Networks

The uncertainty estimate of an INN is given by the width of the prediction interval, i.e., $\varvec{u}(\varvec{z}) = \overline{\varvec{\varPhi }}(\varvec{z}) - \underline{\varvec{\varPhi }}(\varvec{z})$. In terms of computational overhead, INNs scale linearly in the cost of evaluating the underlying prediction DNN with a constant factor 2. In contrast, the popular MCDrop [10] scales linearly with a factor *T* which is proportional to the number of stochastic forward passes and at least $T=10$ is recommended by the authors, see “Baseline UQ methods” section.

Further, INNs come with theoretical coverage guarantees that can be derived from the Markov inequality: Assuming that the loss (3) is optimized during training to yield an INN with vanishing expected gradient with respect to the data distribution, we obtain

4 $$\begin{aligned} \mathbb {P}_{(\varvec{z},\varvec{x})}\left[ \underline{\varvec{\varPhi }}(\varvec{z})_i-\lambda \beta< \varvec{x}_i < \overline{\varvec{\varPhi }}(\varvec{z})_i+\lambda \beta \right] \ge 1-\frac{1}{\lambda }, \end{aligned}$$

for any $\lambda > 0$. In other words, for input and target pair $(\varvec{z},\varvec{x})$ the probability of any component of the target lying inside the predicted interval enlarged by $\lambda \beta$ is at least $1-\frac{1}{\lambda }$. As $\beta$ is usually very small, this ensures a fast decay of the probability of the components of $\varvec{x}$ lying outside the predicted interval bounds. Consequently, a component with a small uncertainty score was correctly reconstructed up to small error with a high probability. Of course, the training distribution needs to be well representative of the true data distribution to extrapolate this property to unseen data.

Finally, the optimization of the loss (3) yields additional information: If the prediction $\varvec{\varPhi }(\varvec{z})$ lies closer to one boundary of the predicted interval, the true target $\varvec{x}$ has a higher probability of lying on the other side of the interval. Consequently, INNs can provide directional uncertainty scores. A quantitative assessment of this capability is given in Fig. 3c+d. We note that it is also possible to explore asymmetric uncertainty estimates in the probabilistic setting, e.g., via exponential family distributions [29] or quantile regression [24]. In contrast to INNs, these methods cannot be applied post hoc as they require substantial modifications to the underlying prediction network.

### Baseline UQ methods

In addition to our INN approach, we consider two other related and popular UQ baseline methods for comparison. First, Monte Carlo dropout (MCDrop) [10] obtains uncertainty scores as the sample variance of multiple stochastic forward passes of the same input signal. In other words, if $\varvec{\varPhi }_1,\dots ,\varvec{\varPhi }_T$ are realizations of independent draws of random dropout masks for the same underlying network $\varvec{\varPhi }$, the component-wise uncertainty estimate is $\varvec{u}_{{\textsc {MCDrop}}}(\varvec{z}) = (\tfrac{1}{T-1} ( \sum _{t=1}^T \varvec{\varPhi }_t(\varvec{z})^2- \tfrac{1}{T} (\sum _{t=1}^T \varvec{\varPhi }_t(\varvec{z}))^2 ))^{1/2}$. Second, a direct variance estimation (ProbOut) was proposed in [22] and later expanded in [12]. Here, the number of output components of the prediction network is doubled and trained to approximate the mean and variance of a Gaussian distribution. The resulting network $\varvec{\varPhi }_{{\textsc {ProbOut}}}:\mathbb {R}^n\rightarrow \mathbb {R}^n\times \mathbb {R}^n, \varvec{z}\mapsto (\varvec{\varPhi }_{\text {mean}}(\varvec{z}), \varvec{\varPhi }_{\text {var}}(\varvec{z}))$ is trained by minimizing the empirical loss $\sum _i \Vert (\varvec{y}_i-\varvec{\varPhi }_{\text {mean}}(\varvec{z}_i)) / \sqrt{\varvec{\varPhi }_{\text {var}}(\varvec{z}_i)}\Vert _2^2 + \Vert \log \varvec{\varPhi }_{\text {var}}(\varvec{z}_i)\Vert _1$. The component-wise uncertainty score of ProbOut is $\varvec{u}_{{\textsc {ProbOut}}}(\varvec{z}) = (\varvec{\varPhi }_{\text {var}}(\varvec{z}))^{1/2}$. Note that, in contrast to INN and MCDrop, the ProbOut approach requires the incorporation of UQ already during training. Thus, it cannot be employed as a post hoc evaluation of an already trained, underlying network $\varvec{\varPhi }$. The role of the actual prediction network is taken by $\varvec{\varPhi }_\text {mean}$.

## Experiments

We present experiments for two different inverse problems. First, a deconvolution task with 1D signals, and second a tomography task on real-world 2D image signals. Both setups are described in more detail below. The description of all hyperparameters for the experiments is kept brief and we refer to our publicly available code at https://github.com/luisoala/inn for full details.

**Fig. 2:**
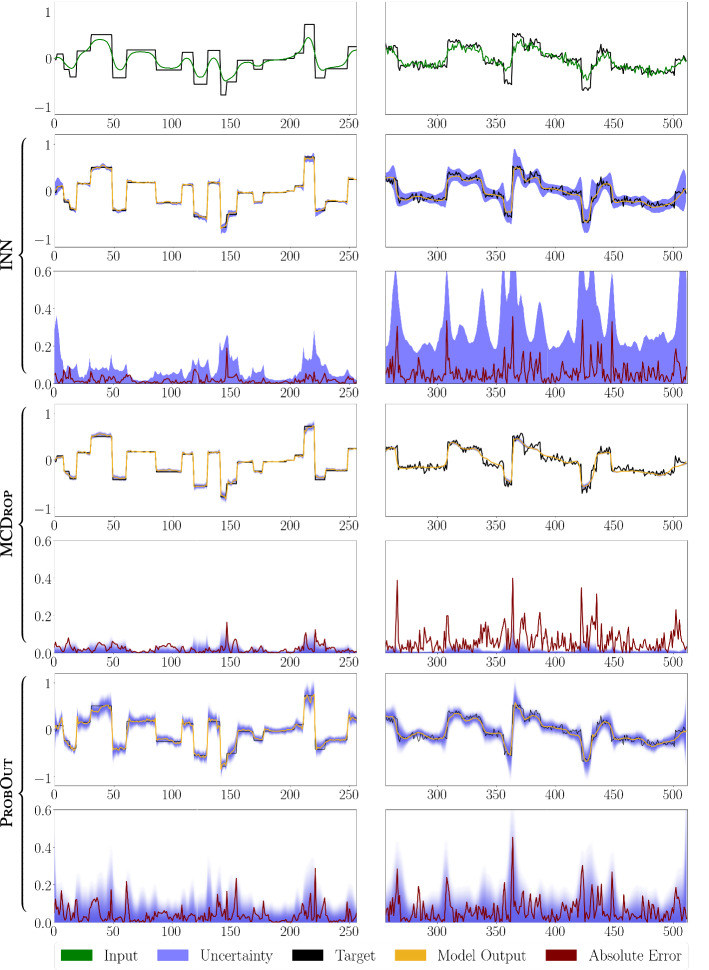
Results for the deconvolution task for one exemplary signal without noise (left) and with additive Gaussian noise ($\sigma =0.05$) on both the measurements $\varvec{y}$ and signal $\varvec{x}$ (right). The first row shows inputs $\varvec{z}=\varvec{y}$ and targets $\varvec{x}$. Below the target $\varvec{x}$, prediction $\varvec{\varPhi }(\varvec{z})$ and uncertainty score $\varvec{u}(\varvec{z})$ as well as the uncertainty compared to the absolute error $|\varvec{\varPhi }(\varvec{z})-\varvec{x}|$ are shown for the three UQ methods.

### Case study A: deconvolution of 1D signals

We start with a synthetic, didactic experiment, inspired by a one-dimensional deconvolution task, to demonstrate the properties of INNs discussed in “Properties of Interval Neural Networks” section. For this purpose, we choose $n=m=512$ and $\varvec{A}= \varvec{D}^\top \varvec{S}\varvec{D}$, where $\varvec{D}$ is a discrete cosine transform (Type I DCT) and $\varvec{S}$ is a diagonal matrix with entries $s_j = \left( \frac{n-j}{n-1}\right) ^\nu \in [0,1]$, that decay with a fixed exponent $\nu =8$. We draw synthetically generated signals $\varvec{x}$ from a distribution of piecewise constant functions with random jump positions and heights, see Fig. 2. The corresponding measurements $\varvec{y}$ are computed according to (1). We generate a data set consisting of 2000 sample pairs $(\varvec{y}_i, \varvec{x}_i)$, 1600 of which were used for training, 200 for validation and 200 for testing. The underlying prediction network $\varvec{\varPhi }$ is a convolutional neural network (consisting of ten convolutional layers and three dropout layers in between) trained to directly map $\varvec{y}$ to $\varvec{x}$, i.e., we use $\varvec{A}^\dagger =\mathrm {Id}$ and thus $\varvec{z}=\varvec{A}^\dagger \varvec{y}=\varvec{y}$ in this experiment. We trained the underlying network $\varvec{\varPhi }$ for 100 epochs using Adam [15]. The interval parameters of the INN were subsequently trained for another 100 epochs with $\beta =2\cdot 10^{-3}$. For the MCDrop comparison, we use $T=64$ samples. The ProbOut model was trained in the same way as $\varvec{\varPhi }$ using 100 Adam epochs. Note that all subsequent evaluations, as well as the plots in Fig. 2 are computed using test samples.

**Fig. 3:**
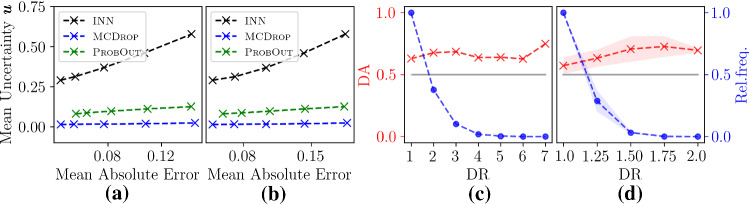
**a** Mean uncertainty of the three UQ methods for varying levels $\sigma$ of additive Gaussian on the measurements $\varvec{y}$ for the deconvolution task. **b** Corresponding results for additive noise on both the measurements $\varvec{y}$ and signals $\varvec{x}$. (c) Illustration of the directional information contained in the INN output intervals for the deconvolution task. The additional right axis (in blue) displays the relative frequency of signal components for each directionality ratio. (d) Corresponding results for the CT task. The mean and standard deviation across three independent complete experimental runs are shown.

In order to evaluate the UQ methods’ abilities to capture uncertainty due to noisy data, we consider additive Gaussian noise $\varvec{\eta }\sim \mathcal {N}(\varvec{0},\sigma ^2\cdot \mathrm {Id})$ on the measurements over a range of noise levels $\sigma$ (Fig. 3a) as well as $\varvec{\eta }_1,\varvec{\eta }_2\sim \mathcal {N}(\varvec{0},\sigma ^2\cdot \mathrm {Id})$ on the measurements and targets, where (1) is adjusted to $\varvec{y}=\varvec{A}(\varvec{x}+\varvec{\eta }_1)+\varvec{\eta }_2$ (Fig. 3b and right column of Fig. 2). In this case, INNs are able to capture the additional uncertainty of $\varvec{\eta }_1$ using the bias parameters of the final network layer. In Fig. 3, it can be observed how in contrast to MCDrop, our method and ProbOut are able to capture independent noise in the data with ProbOut reacting to a lesser degree than the INN. Note also that in Fig. 3 some of the ProbOut evaluations are shifted to the right, indicating a reduced reconstruction performance compared to the other methods.

Finally, we determine the directional information of the INN uncertainty scores as discussed in “Properties of Interval Neural Networks” section. For this, we define the component-wise *directionality ratio* by $\mathrm {DR}(\varvec{z}) = \max \{\overline{\varvec{\varPhi }}(\varvec{z}) -\varvec{\varPhi }(\varvec{z}),\varvec{\varPhi }(\varvec{z})-\underline{\varvec{\varPhi }}(\varvec{z})\} / {\min \{\overline{\varvec{\varPhi }}(\varvec{z})-\varvec{\varPhi }(\varvec{z}),\varvec{\varPhi }(\varvec{z})-\underline{\varvec{\varPhi }}(\varvec{z})\}}$, i.e., as the ratio between the larger and smaller part of the interval $[\underline{\varvec{\varPhi }}(\varvec{z}), \overline{\varvec{\varPhi }}(\varvec{z})]$ when divided by the prediction $\varvec{\varPhi }(\varvec{z})$. The *directionality accuracy* (DA) is the relative frequency of target components corresponding to a given DR that are contained in the larger interval part. As displayed in Fig. 3c, d, INNs achieve a DA consistently above 0.5 (chance), indicating that the interval uncertainty scores contain directional information.

### Case study B: limited angle computed tomography

Next, we consider a 2D computed tomography (CT) task on real-world data in order to evaluate the detection capabilities of the UQ methods with respect to the three failure modes (i)–(iii). More precisely, we consider limited angle CT, which has applications in dental tomography, breast tomosynthesis or electron tomography. For this, $\varvec{A}$ is a subsampled discrete Radon transform with subsampling corresponding to a moderate missing wedge of $30^\circ$. Limited angle measurements are simulated according to (1) and the non-learned inversion $\varvec{A}^\dagger$ is based on the filtered backprojection algorithm (FBP) [21]. The underlying prediction network is a U-Net [25] variant. Our experiments are based on a data set consisting of $512\times 512$ human CT scans from the AAPM Low Dose CT Grand Challenge data [20].^1^ In total, it contains 2580 full-dose images with a slice thickness of 3mm from 10 patients. Eight of these ten patients were used for training (2036 samples), one for validation (214 samples) and one for testing (330 samples). We trained the underlying network $\varvec{\varPhi }$ for 400 epochs using Adam [15]. The interval parameters of the INN were subsequently trained for another 15 epochs with $\beta = 10^{-4}$. We limited the interval training to the last twelve layers. For the MCDrop comparison, we use $T=128$ samples. The ProbOut model was trained in the same way as $\varvec{\varPhi }$ using 400 Adam epochs.

#### Experiment B (i): general prediction error detection

**Fig. 4:**
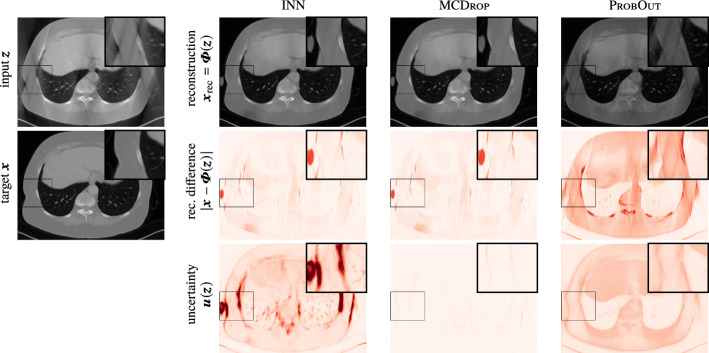
Results of three UQ methods for the Error Detection experiment for one exemplary data sample of the limited angle CT task. The plotting windows are equally adjusted for better contrast.

First, we evaluate how helpful UQ scores are for estimating the prediction error caused by the ill-posedness of the challenging CT task, see Fig. 4. The wedge of missing angles in the measurements results in reconstruction artifacts especially at vertical edges in the images. In order to best visualize these geometric effects of the very structured null-space of the limited angle CT forward operator, we do not add noise in this experiment. INNs are clearly able to reveal the reconstruction uncertainty along the “missing edges.” For a more quantitative comparison of the UQ methods, we use the *performance weighted correlation coefficient* $\mathrm {PWCC}(\varvec{z}, \varvec{x}) = \mathrm {corr}(|\varvec{\varPhi }(\varvec{z})-\varvec{x}|, \varvec{u}(\varvec{z})) / \Vert \varvec{\varPhi }(\varvec{z})-\varvec{x}\Vert _2^2$ between the uncertainty score $\varvec{u}$ and the absolute prediction error. Performance weighting (normalizing by the mean squared error of the prediction) is necessary to discourage rewards for poor prediction models with high uncertainties everywhere. The average results over the test set for three independent complete experimental runs are summarized in Table 1. Both INNs and MCDrop are able to detect prediction errors, with INNs achieving slightly higher correlations. In Fig. 3d, the directional accuracy of the INN is illustrated analogously to the corresponding experiment in “Case study A: deconvolution of 1D signals” section. Again it is consistently above 0.5 (chance).

**Table 1:** Mean test results (± standard deviation) averaged over three experimental runs

UQ method	AdvDetect	ArtDetect	ErrDetect	
			PWCC	MSE
INN	$\mathbf {0.56\pm 0.05}$	$\mathbf {0.52\pm 0.03}$	$\mathbf {2211 \pm 403}$	$7.4 \pm 0.65 \times 10^{-4}$
MCDrop	$0.28\pm 0.02$	$0.26\pm 0.01$	$2170 \pm 513$	$7.4 \pm 0.65 \times 10^{-4}$
ProbOut	$0.48\pm 0.12$	$0.34\pm 0.04$	$190 \pm 28$	$6.7 \pm 2 \times 10^{-3}$

Pearson correlation coefficients for the Adversarial Artifact Detection (AdvDetect) and Atypical Artifact Detection (ArtShort) experiments and PWCC with MSE for the Error Detection (ErrDetect) experiment

#### Experiment B (ii): Adversarial Artifact Detection

**Fig. 5:**
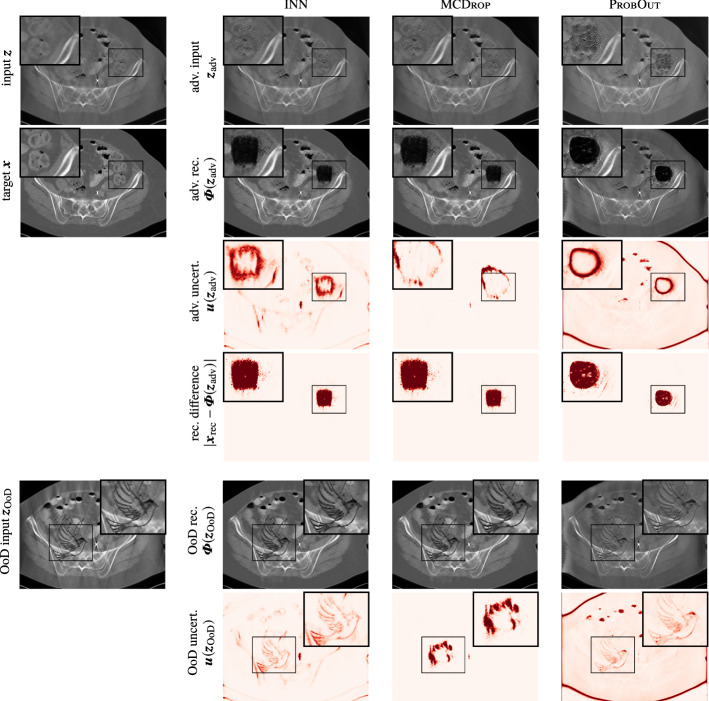
Results of three UQ methods for the AdvDetect and ArtDetect experiments for one exemplary data sample of the limited angle CT task. The plotting windows are equally adjusted for better contrast

Second, we assess the capacity of UQ methods to capture artifacts in the output that were caused by adversarial perturbations. To that end, we create perturbed inputs for each input sample $\varvec{z}$ in the test set by employing the box-constrained L-BFGS algorithm [4] to minimize $\Vert \varvec{\varPhi }(\varvec{z}_{\text {adv}})-\varvec{x}_{\text {adv.~tar.}}\Vert _2^2$ subject to $\varvec{z}_{\text {adv}}\in [0,1]^n$. The adversarial targets $\varvec{x}_{\text {adv.~tar.}}$ are created by subtracting 1.5 times its mean value from $\varvec{x}_{\text {rec}}$ within a random $50\times 50$ square, leading to clearly visible artifacts in the corresponding reconstructions; see Fig. 5. It is arguable, whether the technical aspects of such an adversarial perturbation (i.e., attacking subsequently to a model-based inversion) is a realistic scenario in the context of inverse problems. However, for our purposes, such a simple setup (see also [13]) is sufficient. We refer to [1], where adversarial noise is mapped to the measurement domain. In order to assess the detection capacity for this failure mode, the different UQ schemes are then used to produce uncertainty heatmaps for the generated adversarial inputs. A quantitative evaluation is carried out by computing the mean Pearson correlation coefficient between the pixel-wise change in the uncertainty heatmaps $|\varvec{u}(\varvec{z})-\varvec{u}(\varvec{z}_{\text {adv}})|$ and the change of reconstructions $|\varvec{x}_{\text {rec}}-\varvec{\varPhi }(\varvec{z}_{\text {adv}})|$. The results are summarized in Table 1 and illustrated in Fig. 5. We observe that both INN and ProbOut are able to detect the image region of adversarial perturbations, with INN achieving the highest correlation. This shows that both methods are able to visually highlight the effect that visually almost imperceptible input perturbations can have on the reconstructions.

#### Experiment B (iii): Atypical Artifact Detection

The third experiment is designed analogous to the setup described by [1], i.e., an atypical artifact, which was not present in the training data, is randomly placed in the input to produce $\varvec{z}_{\text {OoD}}$. More precisely, the silhouette of a peace dove is inserted in each image of the test set; see Fig. 5. The simulation of the measurements and model-based inversions is carried out as before. A quantitative evaluation is carried out by computing the mean Pearson correlation coefficient between the change in the uncertainty heatmaps $|\varvec{u}(\varvec{z})-\varvec{u}(\varvec{z}_{\text {OoD}})|$ and a binary mask marking the region of change in the inputs. This evaluation isolates the uncertainty caused by atypical artifacts and allows us to verify in a controlled manner how the uncertainty scores of each UQ method react to the artifacts. During deployment, such controlled isolation is not possible. Instead, the joint uncertainty heatmaps $\varvec{u}(\varvec{z}_{\text {OoD}})$ will also capture other sources of uncertainty, thus providing a more comprehensive alarm system. The results are summarized in Table 1 and illustrated in Fig. 5. All three UQ methods are correlated with the input change; however, INN again achieves the highest correlation. This shows that UQ in general, and INNs in particular, can serve as a warning system for inputs containing atypical features that might otherwise lead to unnoticed and possibly erroneous reconstruction artifacts.

## Conclusion

We introduced INNs as a deterministic, post hoc and fast approach for computing upper and lower bounds and subsequently uncertainty maps for pre-trained neural networks. We demonstrated that UQ in general and INNs in particular can be used to provide a fine-grained detection of failure modes of image reconstruction DNNs. INNs are able to capture uncertainty due to noise and can be used to obtain directional information. They perform well as an alarms system for errors due ill-posedness, adversarial noise and atypical artifacts and thus offer a promising tool to expose the weaknesses of deep image reconstruction models.

## Footnotes

1 See: https://www.aapm.org/GrandChallenge/LowDoseCT/. We would like to thank Dr. Cynthia McCollough, the Mayo Clinic, and the American Association of Physicists in Medicine as well as the grants EB017095 and EB017185 from the National Institute of Biomedical Imaging and Bioengineering for providing the AAPM data.
